# Influence of maternal weight gain on birth weight: a gestational diabetes cohort

**DOI:** 10.20945/2359-3997000000009

**Published:** 2018-01-01

**Authors:** Livia S. Mastella, Letícia S. Weinert, Vanessa Gnielka, Vânia N. Hirakata, Maria Lúcia R. Oppermann, Sandra P. Silveiro, Angela J. Reichelt

**Affiliations:** 1 Universidade Federal do Rio Grande do Sul Universidade Federal do Rio Grande do Sul Porto Alegre RS Brasil Programa de Pós-Graduação em Ciências Médicas: Endocrinologia, Universidade Federal do Rio Grande do Sul (UFRGS), Porto Alegre, RS, Brasil; 2 Hospital de Clínicas de Porto Alegre Unidade de Bioestatística Porto Alegre RS Brasil Unidade de Bioestatística, Hospital de Clínicas de Porto Alegre (HCPA), Porto Alegre, RS, Brasil; 3 Hospital de Clínicas de Porto Alegre Serviço de Obstetrícia e Ginecologia Porto Alegre RS Brasil Serviço de Obstetrícia e Ginecologia, Hospital de Clínicas de Porto Alegre (HCPA), Porto Alegre, RS, Brasil; 4 Hospital de Clínicas de Porto Alegre Serviço de Endocrinologia Porto Alegre RS Brasil Serviço de Endocrinologia, Hospital de Clínicas de Porto Alegre (HCPA), Porto Alegre, RS, Brasil

**Keywords:** Gestational diabetes mellitus, weight gain, birth weight

## Abstract

**Objective:**

Our objective was to evaluate gestational weight gain (GWG) patterns and their relation to birth weight.

**Subjects and methods:**

We prospectively enrolled 474 women with gestational diabetes mellitus (GDM) at a university hospital (Porto Alegre, Brazil, November 2009-May 2015). GWG was categorized according to the 2009 Institute of Medicine guidelines; birth weight was classified as large (LGA) or small (SGA) for gestational age. Adjusted relative risks (aRRs) and 95% confidence intervals (95% CIs) were determined.

**Results:**

Adequate GWG occurred in 121 women [25.5%, 95% CI: 22, 30%]; excessive, in 180 [38.0%, 95% CI: 34, 43%]; and insufficient, in 173 [36.5%, 95% CI: 32, 41%]. In women with normal body mass index (BMI), the prevalence of SGA was higher in those with insufficient compared to adequate GWG (30% vs. 0%, p < 0.001). In women with BMI ≥ 25 kg/m^2^, excessive GWG increased the prevalence of LGA [aRR 2.58, 95% CI: 1.06, 6.29] and protected from SGA [aRR 0.25, 95% CI: 0.10, 0.64]. Insufficient vs. adequate GWG did not influence the prevalence of SGA [aRR 0.61, 95% CI: 0.31, 1.22]; insufficient vs. excessive GWG protected from LGA [aRR 0.46, 95% CI: 0.23, 0.91].

**Conclusions:**

One quarter of this cohort achieved adequate GWG, indicating that specific ranges have to be tailored for GDM. To prevent inadequate birth weight, excessive GWG in women with higher BMI and less than recommended GWG in normal BMI women should be avoided; less than recommended GWG may be suitable for overweight and obese women.

## INTRODUCTION

Gestational diabetes mellitus (GDM) is typically diagnosed approximately 24-28 weeks using an oral glucose tolerance test (1). Adverse outcomes associated with GDM include increased risk of maternal hypertensive disorders and cesarean section as well as perinatal risks of macrosomia, shoulder dystocia and hypoglycemia (2).

Maternal obesity contributes to GDM and, in an independent fashion, to many other adverse maternal pregnancy outcomes, including pregnancy hypertensive disorders, cesarean section, weight retention, and postpartum diabetes. Adverse outcomes for offspring, including congenital anomalies, macrosomia and indicated preterm delivery, are also increased (3). Excessive weight gain *per se* contributes to an increased prevalence of large for gestational age (LGA) and macrosomia (4).

In 2009, the American Institute of Medicine (IOM) updated their recommendations on weight gain in pregnancy without a specific recommendation for GDM (4). Weight gain has been evaluated in several GDM cohort studies, with variable frequencies of adequate, insufficient or excessive weight gain being reported (5-8).

The objectives of this study were to evaluate how the 2009 IOM recommendations on gestational weight gain (GWG) applied to a contemporary cohort of GDM pregnancies and how the patterns of GWG in GDM impacted birth weight.

## SUBJECTS AND METHODS

We studied a cohort of 508 women with GDM with singleton pregnancies, with at least one prenatal appointment, who delivered at Hospital de Clínicas de Porto Alegre (HCPA), a university hospital. HCPA is located in the Southern state of Brazil, Rio Grande do Sul (population ~11 million inhabitants) and provides medical care through the *Sistema Único de Saúde* (SUS), the national health system. In 2015, more than 600,000 general consultations were performed; approximately 4,000 babies were delivered; and the cesarean rate was 32.8% (9). From November 2009 to May 2015, all eligible women referred from primary care units were consecutively included; a multidisciplinary team provided prenatal care. Women gave their consent after being fully informed, and the authors signed the confidentiality document for data use. The hospital ethics committee approved the study protocol (number 2010-0364). We followed the STROBE statement for the study report (10).

Thirty-four women were excluded: one due to an abortion, one for having congenital achondroplasia and 32 due to missing data on maternal weight or infant birth weight. GDM was diagnosed with a 75-g oral glucose tolerance test (OGTT) using the criteria of fasting plasma glucose ≥110mg/dL or 2-h plasma glucose ≥140mg/dL in 232 women (49%) (11). After 2010, the International Association of Diabetes and Pregnancy Study Groups (IADPSG) recommendation was adopted; 242 (51%) of the women met these criteria (12).

Pregestational weight was self-reported, and prepregnancy BMI was classified according to the World Health Organization criteria (13). Weight and height were measured with light clothes and no shoes. All women were prescribed a normocaloric diet, emphasizing the intake of low glycemic index carbohydrates and fiberrich food. Capillary glucose targets were ≤95mg/dL for pre-meal and ≤120mg/dL for 2-h postprandial measures (14). If goals were not met after 2 weeks of nutritional therapy, pharmacological treatment was initiated. Data on pregnancy evolution, delivery, and maternal and newborn outcomes were obtained from hospital registries.

Ethnicity was self-reported. Schooling was categorized as ≤ 11 years or > 11 years. Total weight gain was the weight measured at admission to delivery minus the pre-pregnancy weight; weight gains until diagnosis and from diagnosis to delivery were calculated. The 2009 IOM guidance on GWG by BMI was used, including the following ranges: underweight women, 12.5 to 18kg; normal weight, 11.5 to 16kg; overweight, 7 to 11kg and obese, 5 to 9kg (4). An A1C test was measured at booking (initial A1C test) and during the 3^rd^ trimester (3^rd^ trimester A1C test). Pregnancy-related hypertension disorders included any diagnosis of gestational hypertension or preeclampsia/eclampsia (15), and the composite of maternal risk factors included hypertensive disorders of pregnancy plus smoking.

Newborns were classified as small for gestational age (SGA) or as LGA according to the Alexander birth weight chart (16), which is routinely used at our hospital. Macrosomia was defined as a term birth weight ≥ 4,000g, and preterm birth was defined as a delivery before the completion of 37 gestational weeks (17).

Plasma glucose was measured by the enzymatic method and an A1C test using HPLC (Variant II Turbo HbA1C, BioRad Laboratories, Hercules, CA, USA, aligned to DCCT recommendations).

### Statistical analysis

We described the prevalence of adequate gestational weight gain as n (%) and evaluated the 95% confidence intervals [95% CIs]. Student's t test, χ^2^ test, ANOVA, and Pearson correlation were applied as appropriate.

Relative risks (RR) and 95% CIs for SGA and LGA were determined according to maternal weight gain within normal or elevated BMI ranges (≥ 25 kg/m^2^). Skin color, living with a partner, schooling, gravidity, weight gain grouped by IOM category, fasting glucose at the time of the diagnostic test, use of pharmacologic treatment, 3^rd^ trimester A1C test, and the composite of maternal risk factors were explored in univariable and multivariable models for SGA. For LGA analysis, we added family history of diabetes to the models. Poisson regression with robust estimation was employed in multivariable analyses. Variables were included if a p value of at least < 0.10 was obtained in univariable analysis or if considered clinically important (gravidity and maternal risk factor).

We used SPSS software version 18.8 for statistical analyses. Statistical significance was set at 0.05, two-sided.

## RESULTS

There were some slight differences in baseline clinical characteristics between women classified by the two GDM diagnostic criteria (Table 1). We analyzed them as a single group because we assumed that dissimilarities were related to distinct profiles captured by each criterion. The main differences were observed for a family history of diabetes (54.3% vs. 42.6%, p = 0.011), pregestational BMI (29.4 ± 6.5 vs. 30.7 ± 7.0 kg/m^2^, p = 0.046); the 2-hour glucose in the diagnostic OGTT (170.7 ± 29.1 vs. 148.5 ± 37.1 mg/dL, p < 0.001); the baseline A1C value (5.7% ± 0.8% vs. 5.4% ± 0.6%, p < 0.001), the latter measurement being within the range of laboratory references (6.0%); and weight at delivery (84.0 ± 17.3 vs. 89.1 ± 18.5 kg, p = 0.002). No differences were found regarding key maternal and perinatal outcomes.

**Table 1:** Comparison of women with gestational diabetes according to two diagnostic criteria

Characteristic/outcome			Brazilian criteria N = 232	IADPSG criteria N = 242	p
			Mean ± SD or %	Mean ± SD or %	
Maternal					
	Age (years)		31.4 ± 6.2	31.2 ± 6.7	0.755
	White ethnicity		75.4	78.9	0.365
	Education (> 11 years)		49.6	47.5	0.656
	With partner		63.4	45.5	< 0.001
	Pregnancies (n)		2.7 ± 1.6	2.8 ± 1.7	0.789
	Current smoker		0.9	3.3	0.064
	Family history of diabetes		54.3	42.6	0.011
	Previous GDM		13.4	13.2	0.964
	Pre-pregnancy BMI (kg/m^2^)		29.4 ± 6.5	30.7 ± 7.0	0.046
	Systolic BP (mmHg)*		115.8 ± 11.9	117.0 ± 12.3	0.287
	Diastolic BP (mmHg)*		72.5 ± 10.2	73.0 ± 9.8	0.591
Glycemia in the OGTT (mg/dL)**					
	Fasting		100.0 ± 27	97.2 ± 16.4	0.177
	1 hour (n = 180)		–	178.4 ± 35.8	–
	2 hour		170.7 ± 29.1	148.5 ± 37.1	< 0.001
			n = 226	n = 200	
2^nd^ trimester A1C test (%)***			5.7 ± 0.8	5.4 ± 0.6	< 0.001
3^rd^ trimester weight gain (kg)			2.0 ± 4.0	2.7 ± 4.2	0.066
Weight at delivery (kg)			84.0 ± 17.3	89.1 ± 18.5	0.002
Total gestational weight gain (kg)			10.5 ± 7.5	10.1 ± 7.9	0.598
Hypertensive disorders of pregnancy			13.8	12.0	0.556
Cesarean section			58.6	52.1	0.151
Offspring					
	Birth weight (g)		3,221.6 ± 578.7	3,246.6 ± 602.8	0.645
	Birth weight category				0.681
		SGA	9.9	12.0	
		AGA	78.9	75.6	
		LGA	11.2	12.4	
	Macrosomia		7.3	8.3	0.704

IADPSG: International Association of Diabetes in Pregnancy Study Groups; GDM: gestational diabetes mellitus; BMI: body mass index; BP: blood pressure; OGTT: oral glucose tolerance test; SGA: small for gestational age; AGA: adequate for gestational age; LGA: large for gestational age.

* n = 230 for the Brazilian criteria and 241 for the IADPSG criteria;

** n = 226 for the Brazilian criteria and 200 for the IADPSG criteria;

*** n = 206 for the Brazilian criteria and 207 for the IADPSG criteria.

Among the 474 women, only one had a BMI < 18.5 kg/m^2^, and this case was analyzed in the normal BMI group; 119 had normal BMIs (n = 120, 25%, 95% CI: 21, 29%); and 354 (75%, 95% CI: 71, 79%) had BMIs ≥ 25 kg/m^2^. Adequate weight gain occurred in 121 women [25.5%, 95% CI: 22, 30%], excessive in 180 [38%, 95% CI: 34, 43%] and insufficient in 173 [36.5%, 95% CI: 32, 41%]. Pre-pregnancy hypertension was present in 12.4% of the women, and preeclampsia/gestational hypertension was present in 9.5% of the women. The average gestational age at delivery was 38±1.5 weeks (range: 30-41 weeks), and the rate of cesarean section was 55.3%. The baseline characteristics of the 474 women according to gestational weight gain categories are presented in Table 2.

**Table 2:** Baseline characteristics of 474 women with gestational diabetes according to the 2009 Institute of Medicine weight gain categories

Characteristic	Weight gain category				p value
	Total n = 474	Insufficient n = 173 (36.5)	Adequate n = 121 (25.5)	Excessive n = 180 (38)	
Age (years)&	31 ± 6.4	33 ± 6.0	31 ± 6.6	30 ± 6.5	0.001
White ethnicity	366 (77)	138 (80)	99 (82)	129 (72)	0.073
Education, > 11 years	230 (49)	84 (49)	57 (47)	89 (49)	0.924
With partner	257 (54)	101 (58)	67 (55)	89 (49)	0.232
Pregnancies (n)	2.7 ± 1.7	2.8 ± 1.6	2.9 ± 1.6	2.6 ± 1.7	0.347
Current smoker	10 (2)	4 (2.3)	2 (1.7)	4 (2.2)	0.920
Family history of diabetes	229 (48)	74 (43)	63 (52)	92 (51)	0.185
Previous GDM	63 (13)	26 (15)	15 (12)	22 (12)	0.699
Pre-pregnancy BMI (kg/m^2^)	30.1 ± 6.8	30.7 ± 7.8	29.2 ± 6.5	29.9 ± 5.8	0.194
Systolic BP (mmHg), n = 471	116 ± 12	115 ± 13	116 ± 12	118 ± 12	0.120
Diastolic BP (mmHg), n = 471	73 ± 10	73 ± 10	71 ± 9	74 ± 11	0.163

GDM: gestational diabetes mellitus; BMI: body mass index; BP: blood pressure.

Data represent the mean ± standard deviation (SD) or n (%). ANOVA and Tukey's test for multiple comparisons χ^2^ test and Z test for proportion with Bonferroni adjustment.

& Insufficient and excessive groups are significantly different.

When demographic and social characteristics across GWG categories (insufficient vs. adequate vs. excessive) were analyzed, maternal age was higher in women with insufficient GWG compared to those with excessive gain (Table 2). Fasting plasma glucose (mg/dL) in the OGTT was available for all women (98 ±18 vs. 98 ±26 vs. 100 ± 23, p = 0.455), 1 h glucose was available for 180 women (182 ±35 vs. 185 ±38 vs. 171 ±34, p = 0.104) and 2 h glucose was available for 426 women (162 ±31 vs. 162 ± 40 vs. 158 ±35, p = 0.558). The initial A1C level was measured in 413 women at a mean gestational age of 31 ± 5.8 weeks and was similar across groups (5.6%±0.7% vs. 5.5%±0.8% vs. 5.7% ±0.8%, p=0.070).

Women who gained insufficient weight were more likely to be receiving pharmacological treatment (insulin or oral agents) compared to those with adequate gain (58% vs. 40%, p = 0.009). Metformin treatment was less frequent in those with adequate weight gain (31%) compared to those with insufficient weight gain (47%, p = 0.023), but in the excessive weight gain group (42%), the rates were not different compared to the two other groups. Insulin use was similar across all three groups (19.0% vs. 13.2% vs. 16.1%, p = 0.407).

The primary data on maternal weight gain by IOM category for the two BMI groups (normal and overweight/obese) are displayed in Table 3. Total weight gain increased significantly across the groups. Weight at delivery increased significantly across the three IOM categories in normal BMI women (63.3±6.4 vs. 69.9±6.7 vs. 80.7±7.3 kg, p =0.001). In women with BMI ≥ 25 kg/m^2^, the average weight gain was almost 10 kg higher in the excessive weight gain group.

**Table 3:** Pregnancy outcomes according to pre-pregnancy body mass index and 2009 Institute of Medicine weight gain categories in 474 women with gestational diabetes

Outcome according to BMI			Weight gain category			p value
			Insufficient	Adequate	Excessive	
**BMI < 25 kg/m^2^ (n = 120)**			**n = 54**	**n = 34**	**n = 32**	
**Maternal**						
	3^rd^ trimester weight gain#		-0.7 ± 4.6	2.4 ± 2.3	4.1 ± 5.3	< 0.001
	Weight at delivery (kg)¶		63.3 ± 6.4	69.9 ± 6.7	80.7 ± 7.3	< 0.001
	Total weight gain (kg)¶		6.8 ± 3.0	13.3 ± 1.3	22.6 ± 5.0	< 0.001
	Hypertensive disorders of pregnancy		7 (13)	2 (6)	2 (6)	0.427
	Cesarean section		24 (44)	18 (53)	16 (50)	0.722
	Maternal risk factors		12 (22)	2 (6)	4 (13)	0.101
**Offspring**						
	Birth weight (g)#		2868 ± 478	3284 ± 368	3413 ± 454	< 0.001
	Birth weight category					< 0.001
		SGA#	16 (30)	0 (0)	1 (3)	
		AGA£	37 (69)	33 (97)	25 (78)	
		LGA&	1 (2)	1 (3)	6 (19)	
**BMI ≥ 25 kg/m^2^ (n = 354)**			**n = 119**	**n = 87**	**n = 148**	
**Maternal**						
	3^rd^ trimester weight gain¶		0.7 ± 2.8	2.2 ± 3.4	4.1 ± 4.3	< 0.001
	Weight at delivery (kg)†		88.0 ± 18.0	88 ± 16.0	98 ± 14	< 0.001
	Total weight gain (kg) ¶		1.8 ± 3.1	8.2 ± 1.8	16.4 ± 5.8	< 0.001
	Hypertensive disorders of pregnancy		11 (9)	12 (14)	27 (18)	0.110
	Cesarean section		66 (56)	49 (56)	89 (60)	0.715
	Maternal risk factors*		35 (29)	23 (26)	43 (29)	0.882
**Offspring**						
	Birth weight (g)†		3157 ± 642	3157 ± 569	3425 ± 586	< 0.001
	Birth weight category					< 0.001
		SGA§	12 (10)	17 (20)	6 (4)	
		AGA	94 (79)	64 (74)	113 (76)	
		LGA§	13 (11)	6 (7)	29 (20)	

* Maternal risk factors include: hypertensive disorders of pregnancy plus smoking.

BMI: body mass index; n: number; SGA: small for gestational age; AGA: adequate for gestational age; LGA: large for gestational age. Data represent the mean ± standard deviation (SD) or n (%).

ANOVA and Tukey's test for multiple comparisons χ^2^ test and Z test for proportion with Bonferroni adjustment.

# insufficient group is significantly different from the adequate or excessive groups;

£ insufficient and adequate groups were different;

& insufficient and excessive groups were different;

† insufficient and adequate groups were different from the excessive group;

¶ all groups were different;

§ adequate and excessive groups were different.

Table 3 depicts offspring outcomes according to GWG categories. The mean ± SD birth weight was 3.234±591g; 242 (51%) newborns were male; 52 (11%) were SGA and 56 (12%) LGA; and 37 (7.8%) were macrosomic. The preterm birth rate was 16.5% and was similar across groups (insufficient, 20%, adequate, 13% and excessive, 16%, p=0.315).

The Pearson correlation (r) between GWG and birth weight in normal BMI women was weak, 0.47 (p<0.001), with a coefficient of determination (r^2^) of 0.22; in the group with overweight/obesity, r was lower, 0.24 (p<0.001), and r^2^ was 0.06. The overall r coefficient was 0.26 (p<0.001), and r^2^ was 0.07.

We could not run univariable analyses in the normal BMI group due to the lack of SGA babies and the presence of only one LGA baby in the adequate gain category (Table 3). Pharmacological treatment, excessive total GWG and excessive gain in the 3^rd^ trimester were all statistically significant in the BMI ≥ 25 kg/m^2^ group and were included in the multivariable model, as were gravidity and 3^rd^ trimester A1C. A maternal risk factor composite was added to SGA model, and the fasting plasma glucose at the OGTT was added to the LGA model.

Analyses were run including the total GWG and 3^rd^ trimester gain separately, with adequate weight gain as the reference category. Comparisons of the effect of insufficient GWG on LGA risk were also performed, with excessive GWG as reference. Overall, we analyzed 328 pregnancies with 34 SGA and 42 LGA infants in multivariable models.

As observed in Table 4, the SGA risk decreased 75% with excessive total GWG and 23% with each kilogram gained during the third trimester but was not enhanced by insufficient weight gain. The LGA risk increased independently with total GWG; each kilogram gained in the 3^rd^ trimester increased the risk by 10%. In addition to weight gain, pharmacological treatment increased the LGA risk in the model with total GWG (RR 2.60, 95% CI: 1.11, 5.81). In the model with 3^rd^ trimester weight gain, the risks were also independently increased with pharmacological treatment (RR 2.38; 95% CI: 1.10, 5.28) and 3^rd^ trimester A1C (RR 1.72; 95% CI: 1.28, 2.31). Both adequate and insufficient weight gain had a protective effect upon LGA risk when compared to excessive weight gain.

**Table 4:** Risk for small and large for gestational age babies in women with gestational diabetes with BMI ≥ 25 kg/m^2^ according to gestational weight gain

Risk factor		Multivariable analysis*	
		aRR [95% CI]	p value
		**Small for gestational age**	
Total GWG (adequate as reference)			
	Excessive	0.25 [0.10,0.64]	0.004^a^
	Insufficient	0.61 [0.31,1.22]	0.161
3^rd^ trimester weight gain (kg)		0.87 [0.81,0.95]	0.001^a^
		**Large for gestational age**	
Total GWG (adequate as reference)			
	Excessive	2.58 [1.06,6.29]	0.037^b^
	Insufficient	1.17 [0.42,3.29]	0.768
3^rd^ trimester weight gain (kg)		1.11 [1.05,1.17]	< 0.001^b^
Total GWG (excessive as reference)			
	Adequate	0.39 [0.16,0.95]	0.037^b^
	Insufficient	0.46 [0.23,0.91]	0.026

aRR [95% CI]: adjusted relative risk (95% confidence interval); GWG: gestational weight gain.

* Poisson regression with robust estimation.

a Adjusted for gravidity, pharmacological treatment, maternal risk factors, and 3^rd^ trimester A1C.

b Adjusted for gravidity, fasting plasma glucose in the oral glucose tolerance test, pharmacological treatment, and 3^rd^ trimester A1C.

## COMMENTS

In this GDM cohort, adequate weight gain was attained by only one quarter of women. Those with normal BMI had increased SGA rates with insufficient weight gain. In women with overweight or obesity, excessive GWG increased the LGA risk and protected from SGA, while there was a trend towards a decreased risk of LGA when GWG was insufficient.

Overweight and obesity were frequent in our cohort (75%), with a rate quite similar to that described for type 2 diabetes pregnancies, 80% (18), reflecting the pattern described in 49.1% of non-pregnant Brazilian women in a recent survey (19). Variable rates of obesity, 17 to 71%, have been reported in GDM cohorts; different study populations and diagnostic criteria may explain this wide range (5-7,20,21). Maternal and offspring outcomes can be worsened if women enter pregnancy in the overweight or obese categories; deleterious effects are further magnified by excessive weight gain (22). The high frequency of excessive GWG found (38%) was expected, since up to 65% women were reported gaining more weight than recommended in several cohort studies in GDM (5,7,8,22). This is not exclusive to GDM pregnancies; a similar rate (32.9%) was observed in women in a large Brazilian cohort (n=2,244) (23). Less than recommended GWG was 33.4% in the latter cohort, close to our rate (36.5%) and to those described in GDM (up to 40% of women) (7,8). The influence of GWG on GDM outcomes may therefore be uncertain, leading to the conclusion by a National Institutes of Health committee that evidence was insufficient “because of inconsistency across studies and imprecise effect estimates” (24).

We observed higher weight gain until GDM diagnosis followed by lower gain thereafter, as described in other studies (25,26); this reinforces the idea that more than intervention *per se*, being labeled GDM may increase treatment compliance (27). Moreover, we suppose that excessive weight might be perceived as deleterious by GDM mothers, since overweight and obese women gained approximately 5 kg less than their normal BMI counterparts along pregnancy. Women with insufficient weight gain in our cohort frequently needed pharmacological treatment; this association was also found in the Atlantic-DIP cohort, where insulin use was common in women with lower weight gain (5), in contrast to what was previously described (25). A possible explanation could be the presence of a more severe degree of metabolic disorder. We could not further explore this possibility because we did not measure insulin or C-peptide. We can speculate that this may in part also explain the interesting finding that women gaining insufficient weight were older than those with excessive weight gain. Another explanation for this latter finding could be ascribed to the presence of some degree of placental insufficiency in the oldest group or to compliance being greater due to their previous life experiences.

Although excessive and insufficient weight gain were frequent, maternal outcomes seemed unaffected in our study, opposing findings described by other authors: a higher weight gain in GDM pregnancies increased risks of cesarean section (7,22) and pregnancy-related hypertension by almost two-fold in Irish women with excessive weight gain (5).

Regarding offspring outcomes, we found that only 7% of birth weight could be explained by maternal GWG. Despite this, normal BMI women who gained less than the recommended GWG delivered more SGA babies, while in overweight/obese women we did not observe this association. Furthermore, the SGA rate was close to that of the LGA rate for the whole group, an unexpected finding. We could speculate that close surveillance of diet and weight gain could eventually be an explanation for both an increased rate of SGA in normal BMI women and a decreased rate of LGA in women with adequate or insufficient weight gain, while in women with excessive GWG, high rates of LGA remained. In non-diabetic pregnancies, delivery of SGA or low birth weight babies (<2,500g) is associated with multiple factors, such as hypertension, smoking and insufficient weight gain (27). No difference in hypertension or smoking rates across the weight gain groups was found. High rates of SGA were not expected, as it is well established that GDM treatment *per se* does not increase this risk (28). However, 22% of birth weight was ascribed to GWG in normal BMI women in our study, which could partially explain our findings. Weight gain below recommendations was not related to increased rates of SGA in other GDM cohorts (8,25) nor was it in a type 2 diabetes cohort (18); of note, the results were not adjusted by pre-pregnancy BMI categories. Weight loss in GDM women with BMI ≥25kg/m^2^ resulted in increased SGA in a large American cohort, despite protecting against LGA and macrosomia, although in the study, the last weight measurement was taken around a mean gestational age of 34.8 weeks (29). The linkage between SGA and poor maternal weight gain, which is stronger in underweight women, is not well established yet for other BMI categories in non-GDM pregnancies and not even in GDM, though it is described in large cohort studies and remains positive when adjusted for confounders such as smoking and hypertension (4). It is tempting to speculate that other factors such as vitamin D deficiency might play a role. Maternal vitamin D deficiency and an increased rate of SGA births have been previously described in our cohort (30).

Excessive birth weight is conditioned by maternal obesity and hyperglycemia as well as by excessive weight gain (21,31,32). The association of birth weight with maternal weight gain has been described in women with type 2 diabetes (18), in obese-only women (31), and in obese GDM women (33), reflecting the independent role of those factors. Gestational diabetes leads to excessive birth weight irrespective of diagnostic criteria (2), while proper treatment has been associated with decreased risk (28). In our cohort, as in others, higher rates of LGA were associated with excessive weight gain mainly in overweight and obese women (7,8,25). In the Atlantic-DIP cohort, adjusted risks of similar magnitude to ours were reported for LGA (5), while in another cohort, LGA was increased in obese, but not in overweight, women (6). It is worthy of consideration that those studies calculated GWG from the time of booking. The independent effects of hyperglycemia and weight gain on birth weight were recently quantified: an A1C test < 5.0% avoided 47% of LGA, while adequate GWG avoided 52% of LGA (32). Finally, total GWG was not the only important factor in our study; weight gain in the third trimester also independently influenced LGA risk. A trend towards LGA risk was previously reported in women with excessive GWG after GDM diagnosis (34).

Insufficient GWG and its influence on LGA have been less commonly evaluated. A tendency toward a decreased LGA risk was previously reported in obese GDM women, as well as in overweight women (aRR 1.05, 95% CI: 0.68, 4.19), with a risk magnitude similar to ours (aRR 1.17 (95% CI: 0.42, 3.29) (6). The protective effect of lower GWG on LGA risk compared to excessive gaining was expected, as we compared two extremes, but it is worth saying that women with insufficient GWG had a trend toward delivering more LGA babies than those gaining adequate weight. We believe that the independent effects of hyperglycemia and increased BMI could prevail over that of GWG because their additive effects, which are mediated through maternal hyperlipidemia and relative insulin insufficiency, further stimulate insulin secretion by the fetal pancreas, promoting intrauterine overgrowth (21). Appropriate treatment strategies, including reduced weight gain, potentially counteract these metabolic effects (35).

The main strength of our study is the possibility to evaluate the 2009 IOM recommendation on GWG in a mixed ethnic cohort with a typical GDM profile of excessive BMI at the beginning of pregnancy. In addition to the well-known effects of excessive GWG, we demonstrated that less than recommended GWG might not be deleterious in GDM pregnancies. A study limitation, the influence of treatment on pregnancy outcomes, is inherent to the study design and is mitigated by the similar antenatal care offered throughout the time period.

In conclusion, only one quarter of this cohort achieved weight gain within the 2009 IOM guidance, perhaps indicating that specific ranges should be tailored for GDM pregnancies. Less than currently recommended GWG should be avoided in normal BMI women, although it may be suitable for overweight and obese women because it prevents excessive birth weight. Excessive GWG should be prevented in overweight and obese women to reduce the risk of large for gestational age babies.
